# Night work during pregnancy and preterm birth—A large register-based cohort study

**DOI:** 10.1371/journal.pone.0215748

**Published:** 2019-04-18

**Authors:** Ina Olmer Specht, Paula E. C. Hammer, Esben M. Flachs, Luise M. Begtrup, Ann D. Larsen, Karin S. Hougaard, Johnni Hansen, Åse M. Hansen, Henrik A. Kolstad, Reiner Rugulies, Anne Helene Garde, Jens Peter Bonde

**Affiliations:** 1 Department of Occupational and Environmental Medicine, Bispebjerg and Frederiksberg Hospital, Copenhagen, Denmark; 2 The Parker Institute, Bispebjerg and Frederiksberg Hospital, Frederiksberg, Denmark; 3 The National Research Centre for the Working Environment, Copenhagen, Denmark; 4 Department of Public Health, University of Copenhagen, Copenhagen, Denmark; 5 Danish Cancer Society Research Center, Copenhagen, Denmark; 6 Department of Occupational Medicine, Danish Ramazzini Centre, Aarhus University Hospital, Aarhus, Denmark; 7 Department of Psychology, University of Copenhagen, Copenhagen, Denmark; Holbæk Hospital, DENMARK

## Abstract

**Background:**

Melatonin stimulates the production of progesterone, which is essential for the maintenance of pregnancy. Since melatonin in blood is reduced due to work under illuminated conditions during night work, it has been hypothesized that night work may increase the risk of preterm birth. Previous meta-analyses have not revealed increased risk of preterm birth in women working night shifts during pregnancy. Still, these studies might have been limited by inaccurate self-reports of timing, intensity and duration of night work most likely causing bias towards the null. The aim of this is study was to investigate if the frequency and duration of night work during the first (week 1–12) and second (week 13–22) trimester of pregnancy were associated with risk of preterm birth when objective and prospective data on night work are used.

**Method:**

In a register-based prospective cohort study, we obtained individual day-to-day information on working hours from The Danish Working Hour Database (DWHD, a payroll database including all public service employees in administrative Danish Regions from 2007–2013) and information on preterm birth from the Danish Medical Birth Registry. Night-shift was defined as at least three working hours between 23:00 and 06:00. Preterm birth was defined as giving birth during gestational weeks 23–37. Odds of preterm birth according to working night shifts were analysed by logistic regression.

**Results:**

We identified 16,501 pregnant women eligible for the study, of which 10,202 women (61.8%) had at least one night-shift during the first 22 gestational weeks. The risk of preterm birth was not elevated among women working night shifts compared to women working only day shifts during either the first or second trimester. Within night-shift workers, the risk was not related to the number of night shifts, the duration of night shifts, consecutive night shifts or quick returns defined as short intervals between shifts. Odds of preterm birth was not related to change of working schedule from the first to second trimester, although women changing from night shifts in the first trimester to day work only in the second trimester displayed a weak increased odds of preterm birth (OR 1.21, 95%CI 0.98–1.49) compared to women working night shifts in both trimesters.

**Conclusion:**

Our results, which are without bias from self-report of either exposure or outcome, are in line with the results of previous meta-analyses. Due to the detailed information on hours worked during pregnancy, we were able to investigate several dimensions of night work not previously investigated, of which none were associated with elevated risk of preterm birth.

## Introduction

Working hours outside what is regarded as regular working hours, including night work, are highly prevalent in both industrialized and post-industrialized societies [1], particularly among health care professionals. As most employees in health care are women [2], many within their reproductive age, there is a concern about possible hazardous effects of night work on the course of pregnancy and fetal development.

During work at night the circadian rhythm is disturbed due to the exposure to light. The circadian rhythm aligns physiological and behavioural rhythms like sleep, appetite, hormone levels, blood pressure, temperature and alertness to the 24-hours day-night cycle. Repeated changes of the 24-hour maternal circadian rhythm may influence fetal development [3] by interfering with development of the offspring’s ability to synchronize its own oscillating circadian rhythms [4]. The disruption of circadian rhythms may blur the tightly coordinated developmental processes, as initiation of specific developmental events is often coordinated by surges in specific hormones [5,6]. Melatonin, for instance, is primarily synthesized and secreted by the pineal gland during the dark period of the diurnal cycle (biological night). Exposure to light during this period suppresses the amplitude of melatonin, which might affect the circadian rhythms, especially if it occurs during several consecutive nights [7]. Melatonin, which is elevated in pregnant women, also possesses antioxidant, free-radical scavenger and anti-inflammatory properties and might have an essential role in reducing oxidative stress during pregnancy [8,9]. Findings in a study in mice furthermore suggest that melatonin may prevent inflammation-associated preterm delivery [10]. Importantly, melatonin stimulates the production of progesterone, which is essential for the maintenance of pregnancy [11,12].

Meta-analyses of the association between night work and preterm birth have not shown an increased risk for preterm birth, e.g. in the meta-analysis by van Melick et al (2013) 11 studies of shift or night work during pregnancy and risk of preterm birth were pooled and yielded a odds ratio of 1.04 (95% CI 0.90–1.20) [13,14,15]. However, some of the included studies have assessed exposure by retrospective questionnaires or interviews and thus the associations might be hampered by inaccurate self-report of the timing and intensity of night work which might bias results towards the null. Furthermore, few studies were sufficiently statistically powered to investigate first time pregnant women. This is an important study population as these are not biased by experiences from previous pregnancies, e.g. by changing of work schedule during early pregnancy if they have had unfortunate experiences with night work in a previous pregnancy [16].

The potential healthy worker effect presents another important methodological limitation [17]. If employees with a general poor health or more pregnancy complications, which is associated with risk of preterm birth [18], change from night work to day work early in pregnancy, the association between shift work and preterm birth will be assessed in a population of healthier women [19]. It is therefore important to investigate, if study participants changing from night to day work have an increased risk of adverse birth outcomes.

The aim of this study was to examine whether the number of night shifts, the duration of night shifts, consecutive night shifts or quick returns defined as intervals between shifts during the first (week 1–12) and second (week 13–22) trimester of pregnancy were associated with an increased risk of preterm birth. Further, to examine whether associations were modified by change of work schedule during pregnancy. This was done using objectively and prospectively collected data on daily working hours and preterm birth.

## Materials and methods

### The danish working hour database

We used the Danish Working Hour Database (DWHD), which is a national database created from administrative payroll data [20]. DWHD includes daily information on individual working hours for more than 250,000 employees in administration and public hospitals in the five Danish administrative regions from January 2007 to December 2015, based on payroll data. Data from DWHD were linked with the Danish Medical Birth Registry [21] by the unique personal identifier assigned to all residents in Denmark.

### Study population

We identified all pregnant women from the DWHD who gave birth at least once between January 2007 and December 2013 (N = 42,485). We excluded women ≤18 or ≥50 years of age (n = 15), multiple pregnancies (n = 2,957), pregnancies conceived in 2006 (n = 6,403), women without registrations of night or day work during the first 22 weeks of gestation (n = 13,382), as well as women working both day, early morning and evening shifts without night shifts (n = 3,337) (S1 Fig). The period of exposure was defined as gestational week (GW) 1–22, based on the definition of preterm birth, i.e. birth occurring during GW 23 to 37. To avoid clustering effects, each woman contributed with her first pregnancy only during the study period.

### Assessment of exposure to night work

Data on exposure was retrieved from the information on individual daily worked shifts including on-call night work in DWHD. For each shift a participant worked, a precise time for the beginning and the end of the shift was given. Shifts lasting at least three hours were defined as day (start time after 06:00 and end time before 21:00), evening (end time after 21:00 and before 02:00), night (any start and end time including working hours between 23:00 and 06:00) and early morning (start time between 03:00 and 06:00) [22]. Night workers were defined as someone working at least one night shift and day workers as someone working at least one day shift without any night-; early morning- or evening shifts during the first 22 GW.

We investigated whether change of work schedule from the first (week 1–12) to the second (week 12–22) trimester, was associated with risk of preterm birth compared to working schedules including at least one night shift in both time periods.

Concerning the effect of the different dimensions of night work we used the exposure variables as described in our previous study, on night work and hypertensive disorders of pregnancy [22]. We grouped the number of night shifts into 1–12 or ≥13 in the first trimester (GW 1–12), and 1–10 or ≥11 in the second trimester (GW 13–22). The number of night shifts was categorized differently in each trimester due to the cut-of at 22 gestational weeks which is before the end of the second trimester. A long night shift was defined by a duration of >8 hours. Consecutive night shifts were categorized as ‘None’ (only single night shifts), 2–3 (at least one spell of 2–3 consecutive night shifts), and ≥4 (at least one spell of ≥4 consecutive night shifts) during the first or second trimester. Quick returns were defined as intervals between shifts lasting <11 hours [23]. Quick return after a night shift was defined as a recovery period of less than 28 hours after a night shift, in line with recent studies with payroll data [22]. Categories of the number of quick returns and quick returns after a night shift were 0, 1–2 and ≥ 3 quick returns in the first trimester and 0, 1 and ≥2 in the second trimester, corresponding to at least one quick return per month in the highest category.

### Assessment of the outcome and covariates

The observational unit was the first pregnancy in the study period, defined as a child birth reported in the Danish Medical Birth Registry [21]. The studied outcome was preterm birth, defined as a live birth during GW 23–37, corresponding to a gestation length of 154–259 days [24]. Preterm birth is defined relative to gestational age, which most often is defined by ultra sound early in pregnancy (crown-rump and biparietal diameter), otherwise by the date of the first day in the last menstrual period as reported by the women.

Age (continuous), body mass index (BMI) (<18.5, 18.5–24.9, 25–29.9, ≥ 30 kg/m^2^), parity (1, 2, ≥ 3) and smoking (non-smoker, prior smoker, current smoker) registered by the midwife or family doctor at the first antenatal visit were retrieved from the Danish Medical Birth Registry [25]. Classification of socio economic status (SES) into high, medium or low was based on DISCO-88, the Danish version of the International Standard Classification of Occupations (ISCO-88) [26], in the calendar years 2007–2009 and DISCO-08, the Danish version of ISCO-08 [27], in the calendar years 2010–2013, both accessible from Statistics Denmark. Sickness absence expressed as the sum of all days registered as absence due to sickness lasting at least three hours in DWHD within three months prior to conception date, was categorized as 0, 1–9 and ≥ 10 days.

### Statistical analysis

Using logistic regression, we calculated odds ratios (OR) with 95% confidence intervals (CI) for preterm birth according to the different dimensions of night work (number of night shifts; duration of night shifts; consecutive night shifts; quick returns; and quick returns after a night shift) during gestational weeks 1–12 (first trimester) and 13–22 (second trimester). Analyses were first performed crudely and subsequently adjusted for age, BMI, parity, smoking, SES and sickness absence during the three months prior pregnancy. Analyses investigating duration of night shifts, consecutive night shifts and quick returns were in addition adjusted for the number of night shifts. We performed comparisons of night workers with day workers, and comparisons within night workers, where the night workers with the lowest exposure category served as reference group.

Additionally, we investigated preterm birth according to changes of work schedule from the first to the second trimester using logistic regression, with women working night shifts in both trimesters as the reference, and in a second analysis with women working only days in both trimesters as the reference group.

In sensitivity analyses, the main analyses were repeated for first time pregnant women, to take into account that later pregnancies may be conditional on the outcome of previous pregnancies.

The statistical analyses were conducted on a secure platform at Statistics Denmark using SAS software, version 5.1 for Windows (SAS Institute Inc., Cary, NC, USA). We used a two-sided p-value with a level of significance of 0.05.

### Ethical approval

Through the notification system in the Capital Region of Denmark, the study was approved by the Danish Data Protection Agency (j.nr.: 2012-58-0004). By Danish law, no informed consent is required for a register-based study.

## Results

The study population consisted of 16,501 pregnant women, of which 10,202 women (61.8%) worked at least one night shift during the first 22 gestational weeks (Table 1). Compared to day workers, night workers more often were first time pregnant (54.7% vs. 45.0%, respectively), more often had a medium SES (70.3% vs. 53.9%, respectively) and less often a low SES (7.2% vs. 21.3%, respectively), and were less often current smokers (2.6% vs. 3.6%, respectively). The prevalence of preterm birth was 5.2% among night workers and 5.1% among day workers.

**Table 1:** Characteristics of pregnant workers in public administration and hospitals in Denmark, 2007–2013. N = 16,501.

	Day work ^a^				Night work ^b^			
	n	%	Mean	SD	n	%	Mean	SD
N	6,298	38.2			10,203	61.8		
Age, years	6,298		31.9	4.1	10,203		30.8	3.9
Body Mass Index, kg/m^2^	6,038		23.9	4.6	9,779		23.7	4.3;
Parity								
1	2770	45.0			5,456	54,7		
2	2295	37.3			2,977	29,8		
≥ 3	1096	17.8			1542	15.5		
Smoking during pregnancy								
Non smoker	5,833	92.6			9,462	92.8		
Prior smoker	86	1.4			202	2.0		
Current smoker	223	3.5			262	2.6		
Missing	156	2.5			277	4.9		
Socioeconomic status								
High	1,543	24.9			2287	22.5		
Medium	3,339	53.9			7147	70.3		
Low	1,317	21.3			727	7.2		
Shifts during the first 22 pregnancy weeks								
Day			76.3	22.0			43.1	18.2
Night							12.4	10.0
Evening							10.4	10.6
Early morning							0.03	0.9
Sickness absence three months prior to pregnancy, days			3.0	7.2			2.7	5.7
Sickness absence during the first 22 pregnancy weeks, days			7.0	11.8			6.5	9.1
Preterm birth	319	5.1			528	5.2		

^a^ At least one day shift and no night, evening or early morning shift during the first 22 pregnancy weeks.

^b^ At least one night shift during the first 22 pregnancy weeks.

### Number of night shifts and preterm birth

As presented in Table 2, the number of night shifts during the first and second trimesters showed no associations with preterm birth when night workers were compared to women working only day shifts. Further, no associations were observed when the number of night shifts were analysed among night workers only.

**Table 2:** Odds ratios of preterm birth by number of night shifts during the first and second trimesters among workers in public administration and hospitals in Denmark, 2007–2013. [OR = odds ratio; CI = confidence interval].

Number of night shifts	All women		Cases		Crude		Adjusted^a^	
	N	%	N	%	OR	95% CI	OR	95% CI
1–12 GW (1^st^ trimester)								
0 (Day work ^b^)	6,843	41.5	343	5.0	1	-	1	-
1–12	7,904	47.9	421	5.3	1.07	0.92;1.24	1.03	0.88;1.21
≥ 13	1,754	10.6	83	4.7	0.94	0.73;1.20	0.87	0.66;1.13
13–22 GW (2^nd^ trimester)								
0 (Day work ^b^)	8,845	53,6	469	5.3	1	-	1	-
1–10	6,501	39,4	320	4.9	0.93	0.80;1.10	0.89	0.76;1.04
≥ 11	1,155	7.00	58	5.0	0.94	0.71;1.24	0.91	0.66;1.22
Night workers only ^c^								
1–12 GW (1^st^ trimester)								
1–12	7,904	81.8	421	5.3	1	-	1	-
≥ 13	1,754	18.2	83	4.7	0.88	0.69;1.12	0.84	0.64;1.08
13–22 GW (2^nd^ trimester)								
1–10	6,501	84.9	320	4.9	1	-	1	-
≥ 11	1,155	15.1	58	5.0	1.02	0.76;1.35	1.01	0.73;1.37

^a^ Adjusted for age, body mass index, smoking, socioeconomic status, parity and sickness absence three months prior to pregnancy.

^b^ At least one day shift and no night, evening or early morning shift during the trimester.

^c^ At least one night shift during the trimester.

### Duration of night shifts and preterm birth

The odds of preterm birth was lower during the second trimester in women working night shifts of ≥8 hours compared to women working day shifts only (OR 0.82, 95%CI 0.67–0.99, Table 3). No associations were observed in the analysis of the first trimester, and when the population was restricted to night workers only.

**Table 3:** Odds ratios of preterm birth by duration of night shifts during the first and second trimester among workers in public administration and hospitals in Denmark, 2007–2013. [OR = odds ratio; CI = confidence interval].

Duration of night shifts	All women		Cases		Crude		Adjusted ^a^	
	N	%	N	%	OR	95% CI	OR	95% CI
1–12 GW (1^st^ trimester)								
0 (Day work ^b^)	6,843	41.5	343	5.0	1	-	1	-
≤8 hours^c^	4,761	28.9	248	5.1	1.01	0.85;1.20	1.03	0.86;1.25
>8 hours^d^	4,897	29.7	257	5.4	1.08	0.91;1.27	0.97	0.81;1.17
13–22 GW (2^nd^ trimester)								
0 (Day work ^b^)	8,845	53.6	469	5.3	1		1	
≤8 hours^c^	4,148	25.1	207	5.0	0.94	0.76;1.01	0.97	0.80;1.16
>8 hours^d^	3,507	21.1	171	4.9	0.92	0.79;1.11	**0.82**	**0.67;0.99**
Night workers only^e^								
1–12 GW (1^st^ trimester)								
≤8 hours^c^	4,897	50.7	248	5.1	1	-	1	-
>8 hours^d^	4,761	49.3	256	5.4	1.07	0.89;1.28	0.92	0.75;1.14
13–22 GW (2^nd^ trimester)								
≤8 hours^c^	4,148	54.2	207	5.0	1	-	1	-
>8 hours^d^	3,507	45.8	171	4.9	0.98	0.79;1.20	0.83	0.64;1.04

^a^ Adjusted for age, body mass index, smoking, socioeconomic status, parity and sickness absence three months prior to pregnancy.

^b^ At least one day shift and no night, evening or early morning shift during the trimester.

^c^ At least one night of ≤ 8 hours and no night shifts of ≥ 9 hours.

^d^ At least one night shift of > 8 hours

^e^ At least one night shift during the trimester.

### Consecutive night shifts and preterm birth

When investigating the odds of preterm birth in relation to consecutive night shifts, women working spells of 2–3 consecutive nights had an OR above unity compared to day workers, however statistically significant in the crude analysis only (Table 4). The results were similar in the analysis investigating night workers only.

**Table 4:** Odds ratios of preterm birth by consecutive night shifts during the first or second trimester among workers in public administration and hospitals in Denmark, 2007–2013. [OR = odds ratio; CI = confidence interval].

Number of consecutive night shifts	Women		Cases		Crude		Adjusted^a^	
	N	%	N	%	OR	95% CI	OR	95% CI
1–12 GW (1^st^ trimester)								
0 (Day work ^b^)	6,843	41.5	343	5.0	1	-	1	-
None	3,123	18.9	143	4.6	0.91	0.74;1.12	0.93	0.74;1.17
2–3	4,645	28.2	274	5.9	**1.19**	**1.01;1.40**	1.01	0.92;1.33
≥ 4	1,890	11.5	87	4.6	0.91	0.71;1.16	0.87	0.66;1.12
13–22 GW (2^nd^ trimester)								
0 (Day work ^b^)	8,845	53.6	469	5.3	1	-	1	-
None	2,450	14.9	106	4.3	0.81	0.65;1.00	0.83	0.64;1.06
2–3	3,361	20.4	186	5.5	1.05	0.88;1.24	0.97	0.80;1.17
≥ 4	1,845	11.2	86	4.7	0.87	0.69;1.10	0.83	0.64;1.06
Night workers only ^c^								
1–12 GW (1^st^ trimester)								
None	3,123	32.3	143	4.6	1	-	1	-
2–3	4,645	48.1	274	5.9	**1.31**	**1.06;1.61**	1.18	0.91;1.54
≥ 4	1,890	19.6	87	4.6	1.01	0.76;1.31	0.92	0.66;1.28
13–22 GW (2^nd^ trimester)								
None	2,450	14.9	106	4.3	1	-	1	-
2–3	3,361	20.4	186	5.5	**1.30**	**1.02;1.66**	1.16	0.85;1.61
≥ 4	1,845	11.2	86	4.7	1.08	0.81;1.45	0.98	068;1.42

^a^ Adjusted for age, body mass index, smoking, socioeconomic status, parity and sickness absence three months prior to pregnancy.

^b^ At least one day shift and no night, evening or early morning shift during the trimester.

^c^ At least one night shift during the trimester.

### Quick returns and preterm birth

When investigating quick returns in relation to odds of preterm birth, two or more quick returns during GW 13–22 were associated with lower odds of preterm birth, OR 0.79, 95%CI 0.65–0.95 (Table 5), however this association disappeared after adjusting for the number of night shifts, OR 0.83, 95%CI 0.65–1.05 (data not shown in table). Quick returns after a night shift were not statistically significantly associated with preterm birth (Table 5).

**Table 5:** Odds ratios of preterm birth by number of quick returns ^a^ and quick returns after a night shift ^b^ during the first and second trimester among workers in public administration and hospitals in Denmark, 2007–2013. [OR = odds ratio; CI = confidence interval].

Quick Returns	Women		Cases		Crude		Adjusted^c^	
	N	%	N	%	OR	95% CI	OR	95% CI
1–12 GW (1^st^ trimester)								
0 (Day work ^d^)	6,843	41.5	343	5.0	1	-	1	-
None	3,037	18.4	151	5.0	0.99	0.81;1.20	1.01	0.81;1.25
1–2	2,773	16.8	149	5.4	1.08	0.88;1.31	1.10	0.89;1.36
≥ 3	3,848	23.3	204	5.3	1.06	0.89;1.27	0.93	0.76;1.13
13–22 GW (2^nd^ trimester)								
0 (Day work ^d^)	8,845	53.6	469	5.3	1	-	1	-
None	2,444	14.8	122	5.0	0.94	0.76;1.15	0.95	0.75;1.19
1	1,217	7.4	68	5.6	1.06	0.81;1.36	1.15	0.87;1.40
≥2	3,995	24.2	188	4.7	0.88	0.74;1.05	**0.79**	**0.65;0.95**
Night workers only ^e^								
1–12 GW (1^st^ trimester)								
None	3,037	31.5	151	5.0	1	-	1	-
1–2	2,773	28.7	149	5.4	1.09	0.86;1.37	1.09	0.85;1.41
≥ 3	3,848	39.8	204	5.3	1.07	0.86;1.33	0.93	0.71;1.18
13–22 GW (2^nd^ trimester)								
None	2,444	31.9	122	5.0	1	-	1	-
1	1,217	15.9	68	5.6	1.13	0.83;1.52	1.19	0.85;1.64
≥2	3,995	52.2	188	4.7	0.94	0.75;1.19	0.81	0.61; 1.06
**Quick Returns after a night shift**								
1–12 GW (1^st^ trimester)								
0 (Day work^d^)	6,843	41.5	343	5.0	1.00	-	1.00	-
None	638	3.9	41	6.4	1.30	0.92;1.80	1.26	0.87;1.78
1–2	1,901	11.5	102	5.4	1.07	0.85;1.34	1.01	0.79;1.29
≥ 3	7,119	43.1	361	5.1	1.01	0.87;1.18	0.97	0.82;1.15
13–22 GW (2^nd^ trimester)								
0 (Day work^d^)	8,845	53.6	469	5.3	1	-	1	-
None	371	2.3	19	5.1	0.96	0.58;1.58	0.95	0.55;1.52
1	453	2.7	26	5.8	1.09	0.71;1.60	1.01	0.63;1.54
≥ 2	6,833	41.4	333	4.9	0.91	0.79;1.06	0.88	0.75;1.03
Night workers only^e^								
1–12 GW (1^st^ trimester)								
None	638	6.6	41	6.4	1.00	-	1.00	-
1–2	1,901	19.7	102	5.4	0.83	0.57;1.21	0.79	0.54;1.20
≥ 3	7,119	73.7	361	5.1	0.78	0.56;1.10	0.76	0.54;1.11
13.22 GW (2^nd^ trimester)								
None	371	4.9	19	5.1	1	-	1	-
1	452	5.9	26	5.8	1.13	0.62;2.10	1.06	0.55;2.06
≥ 2	6,833	89.2	333	4.9	0.95	0.61;1.58	0.92	0.57;1.58

^a^ Less than 11 hours between two consecutive shifts.

^b^ Less than 28 hours between a night shift and the consecutive shift.

^c^ Adjusted for age, body mass index, smoking, socioeconomic status, parity and sickness absence three months prior to pregnancy.

^d^ At least one day shift and no night, evening or early morning shift during the trimester.

^e^ At least one night shift during the trimester.

### Work schedule changes and preterm birth

**Fig 1:**
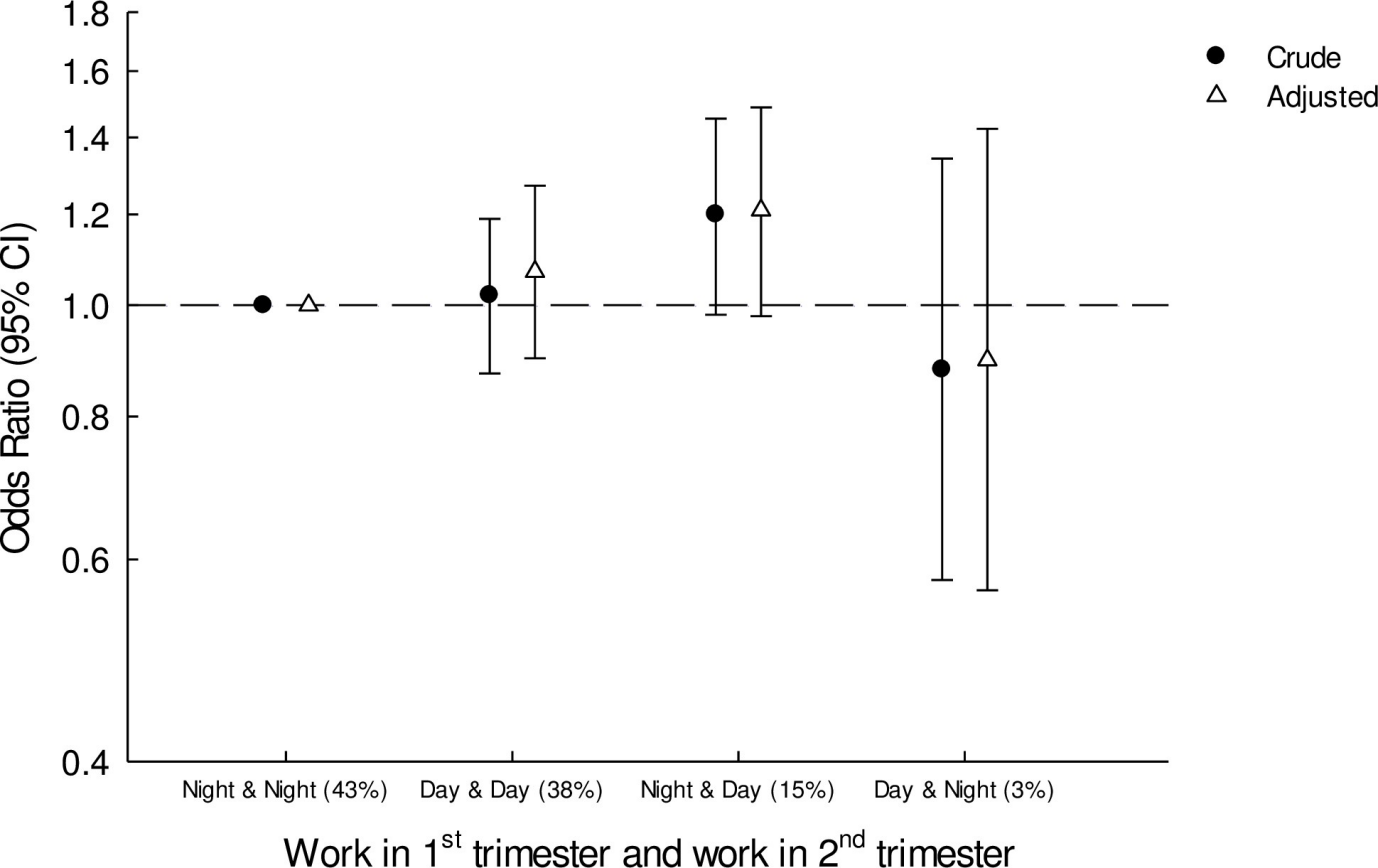
Selection out of/into night work from 1^st^ to 2^nd^ trimesters and odds of preterm birth. **Crude and adjusted analysis.** Adjusted for parity, age of mother, pre-pregnancy BMI, smoking during pregnancy and sick leave 3 months before during pregnancy.

As presented in Fig 1, 43.1% of the women working night shifts during the first trimester also had night work in the second trimester, whereas 15.4% changed from having night work in the first trimester to having only day work in the second trimester. Only 3.3% changed from day work in the first trimester to also having night work in the second trimester. No associations were observed among change in work schedule and odds of preterm birth in either crude or adjusted analysis. Odds ratio of preterm birth was non-significantly increased among workers changing from having night work in the first trimester to having only day work in the second trimester (OR 1.21, 95%CI 0.98–1.49, Fig 1).

We further investigated work schedule changes with day workers as the reference group. Similarly, the odds of preterm birth was non-significantly increased among women changing from having night work in the first trimester to having only day work in the second trimester (OR 1.13, 95%CI 0.91–1.40, S1 Table).

### Sensitivity analyses

Sensitivity analysis of first time pregnant women (N = 8,227) yielded similar results as the main analyses (S2 Table).

## Discussion

In this study, the potential association of night work with risk of preterm birth was investigated with the use of objective and precise daily exposure information on working time. Based on these data we were also able to investigate selection in and out of night work during pregnancy. We found no associations of the number of night shifts, consecutive night shifts or quick returns with preterm birth, after adjustment for covariates. However, women working at least one night shift lasting >8 hours during the second trimester had lower odds of preterm birth compared to day workers.

A reason for our null-findings could be primary selection, i.e. ‘the Healthy Hire Effect’ which proposes that healthier employees are more likely to be selected or self-selected into night work before becoming pregnant [17,19], which we could not assess in our study. We observed a higher prevalence of older, low SES, and currently smoking women among the women working days only as compared to night shift workers, hence the women working night shifts might have been slightly more healthy than day workers. We observed estimates below unity in the majority of the first trimester analyses of highest exposed categories of night work, which support a potential primary selection.

The odds of preterm birth was not related to change of work schedule from the first to the second trimester, i.e. secondary selection which is selection during pregnancy, although data indicated a potential, non-significant increased odds of preterm birth among women changing from night work in the first trimester to day work only in the second trimester compared to women working night shifts in both trimesters. We made two analyses, one with the reference group being women working night shifts in both trimesters and one with a reference group of women working days only in both trimesters with similar results. The argument for using night shift workers as the reference group was that these women might be ‘healthier’, according to the healthy worker survivor effect [17]. In the second analysis, where the reference group consisted of women working days only during both trimesters, we investigated the effect of night work taking the change of working schedule into account. The similar indications of increased risk of preterm birth among women changing from night work to day work in the two analyses, might be due to that this group of pregnant women in general are at a potential higher risk of preterm birth and thus are selected or self-selected out of night work probably due to their health, i.e. a healthy worker survivor effect.

Our study had some limitations. We did not have information on sleeping conditions during on-call night shifts, work load or lighting conditions, nor did we investigate different occupational groups or have the power to specifically investigate permanent night work. However, a prospective study of 40,237 pregnant Danish women within different occupational groups, where 400 had permanent night work, did not find an increased risk of preterm delivery (OR 0.70, 95% CI 0.40–1.23) [28]. A small study did report an increased risk for preterm birth but only in a subgroup of permanent evening or night workers, who stopped working after 24 GW (OR 1.96, 95% CI 1.00–3.83) [29]. Also, information on other hypothetical confounders like fertility treatment, medical conditions, shift work prior to this pregnancy or previous preterm delivery which might affect the ability to work night shifts, were unavailable to this project. All our results were corroborated in the sensitivity analyses only including first time pregnant women. This indicates that the outcome of previous pregnancies did not influence the risk estimates.

Strengths of the study include detailed register information on daily, individual working hours. This enabled the examination of risk according to different dimensions of night work within each gestational trimester as well as the effect of cumulative night work. As the gestational age at delivery was registered in the Danish Medical Birth Registry by the midwives assisting the respective birth, the preterm birth variable was not collected retrospectively or self-reported, hence precluding recall-bias. Even though the study was entirely register-based with limited personal data on health behaviour and lifestyle, though we did include smoking and BMI, we are not aware of important determinants of preterm birth that we were not able to address. Finally, our study was large enough to investigate first time pregnancies.

In conclusion, our results of no increased risk of preterm birth among women working night shifts, are in line with the results of existing meta-analyses, although these might have been biased by self-report and were not able to address selection [14,30]. Due to the detailed information on hours worked during pregnancy, we were able to investigate several dimensions of night work not previously studied, albeit none were associated with increased risk of preterm birth. Finally, our study did not observe that selection out of night work during pregnancy was associated with preterm birth.

## Supporting information

S1 Fig Flow chart of the study population. (TIF)

S1 Table Investigating selection out of/into night work from 1^st^ to 2^nd^ trimesters and odds of preterm birth. **Adjusted analyses**^a. a^ Adjusted for parity, age of mother, pre-pregnancy BMI, smoking during pregnancy and sick leave 3 months before during pregnancy.
(PDF)

S2 Table Sensitivity analysis of first-time pregnant women according to different dimensions of night work and preterm birth. (PDF)
