# Supplementary figures and images for: Night work during pregnancy and preterm birth—A large register-based cohort study
Source: PLoS One. 2019 Apr 18;14(4):e0215748. doi: 10.1371/journal.pone.0215748 (PMC6472821; doi:10.1371/journal.pone.0215748)

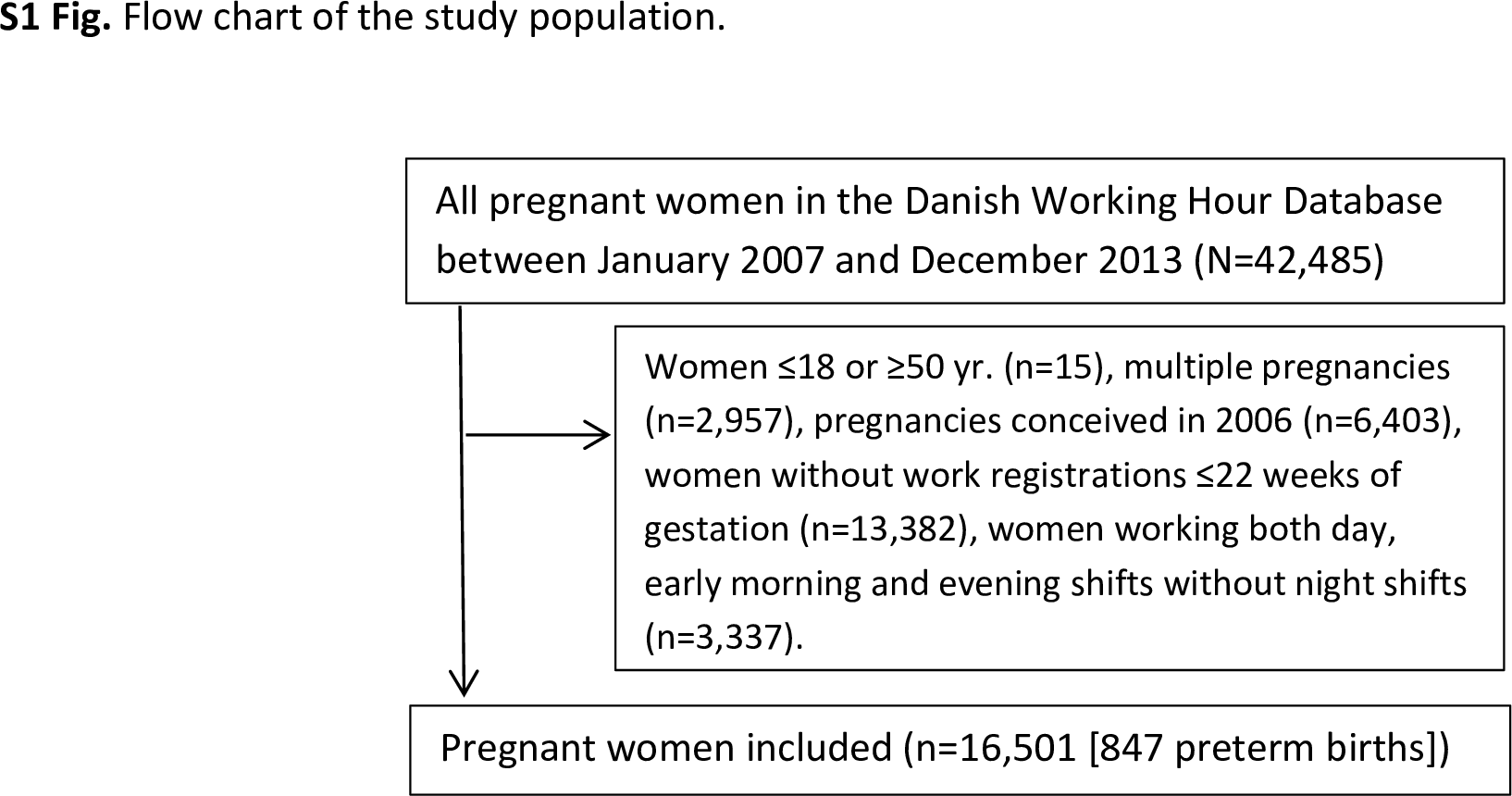

Supplement: S1 Fig — (TIF) [file pone.0215748.s001.tif]
