# Supplementary material for: Night work during pregnancy and preterm birth—A large register-based cohort study
Source: PLoS One. 2019 Apr 18;14(4):e0215748. doi: 10.1371/journal.pone.0215748 (PMC6472821; doi:10.1371/journal.pone.0215748)
Supplement: S1 Table — Adjusted analysesa. a Adjusted for parity, age of mother, pre-pregnancy BMI, smoking during pregnancy and sick leave 3 months before during pregnancy. (PDF) [file pone.0215748.s002.pdf]

S1 Table. Investigating selection out of/into night work from 1st to 2nd trimesters and odds of preterm birth. Adjusted analyses[a].

%	Work in 1st trimester	Work in 2nd trimester	Odds ratio$_a$	95%CL
48.3	Day	Day	1	-
36.1	Night	Night	0.94	0.79; 1.11
12.9	Night	Day	1.13	0.91;1.40
2.8	Day	Night	0.84	0.51; 1.30

[a] Adjusted for parity, age of mother, pre-pregnancy BMI, smoking during pregnancy and sick leave 3 months before during pregnancy.
