# Supplementary material for: Night work during pregnancy and preterm birth—A large register-based cohort study
Source: PLoS One. 2019 Apr 18;14(4):e0215748. doi: 10.1371/journal.pone.0215748 (PMC6472821; doi:10.1371/journal.pone.0215748)
Supplement: S2 Table — (PDF) [file pone.0215748.s003.pdf]

S2 Table. Sub-analysis of first-time pregnant women only (N=8227)

Number of night shifts	Crude OR	95% CI	Adj. OR	95% CI
1-12 GW (1st trimester)				
0 (day work)	1		1	
1-12 night shifts	1.03	0.86-1.25	1.01	0.83-1.24
>= 13 night shifts	0.93	0.68-1.25	0.89	0.63-1.22
13-22 GW (2nd trimester)				
0 (day work)	1		1	
1-10 night shifts	0.88	0.73-1.05	0.87	0.72-1.06
>= 11 night shifts	0.93	0.65-1.30	0.89	0.61-1.28
**Number of consecutive night shifts**				
1-12 GW (1st trimester)				
0 (day work)	1		1	
none	0.86	0.66;1.11	0.92	0.68;1.23
2 to 3	1.12	0.91;1.38	1.05	0.83;1.31
>=4	0.97	0.72;1.29	0.94	0.69;1.28
13-22 GW (2nd trimester)				
0 (day work)	1			
none	0.71	0.53;0.93	0.78	0.55;1.05
2 to 3	0.98	0.79;1.22	0.92	0.73-1.16
>=4	0.92	0.69;1.20	0.90	0.66-1.20
**Night workers only**				
1-12 GW (1st trimester)				
none	1		1	
2 to 3	1.31	1.01-1.71	1.14	0.84-1.55
>=4	1.13	0.81-1.57	1.02	0.71-1.48
13-22 GW (2nd trimester)				
none	1		1	
2 to 3	1.40	1.02-1.93	1.20	0.84-1.73
>=4	1.30	0.90-1.87	1.17	0.78-1.77
**Quick Returns**				
1-12 GW (1st trimester)				
0 (day work)	1		1	
none	0.88	0.67-1.15	0.91	0.68-1.21

1 to 2	1.19	0.93-1.51	1.18	0.91-1.53		
>=3	0.98	0.79-1.22	0.92	0.72-1.17		
13-22 GW (2nd trimester)					incl sum of night shifts	
0 (day work)	1		1		1	
none	0.86	0.65-1.12	0.91	0.67-1.22	0.91	0.64-1.29
1	1.21	0.87-1.64	1.12	0.89-1.72	1.25	0.85-1.80
>=2	0.82	0.66-1.01	**0.77**	**0.61-0.96**	0.77	0.58-1.02
Night workers only						
1-12 GW (1st trimester)						
none	1		1			
1 to 2	1.35	1.00-1.82	1.30	0.95-1.80		
>=3	1.11	0.85-1.48	1.01	0.75-1.38		
13-22 GW (2nd trimester)						
none	1		1			
1	1.41	0.96-2.06	1.37	0.91-2.05		
>=2	0.96	0.71-1.30	0.84	0.61-1.18		
**Quick Returns after a night shift**						
1-12 GW (1st trimester)						
0 (day work)	1		1			
none	1.35	0.88-2.01	1.26	0.79-1.94		
1 to 2	1.07	0.80-1.41	1.04	0.77-1.39		
>=3	0.97	0.80-1.18	0.95	0.77-1.18		
13-22 GW (2nd trimester)						
0 (day work)	1		1			
none	0.93	0.50-1.59	0.99	0.51-1.73		
1	0.81	0.44-1.38	0.73	0.37-1.29		
>=2	0.89	0.74-1.06	0.88	0.72-1.06		
Night workers only						
1-12 GW (1st trimester)						
none	1		1			
1 to 2	0.79	0.50-1.26	0.82	0.51-1.36		
>=3	0.76	0.48-1.10	0.75	0.49-1.20		
13-22 GW (2nd trimester)						
none	1		1			

1	0.87	0.39-1.95	0.74	0.31-1.72
>=2	0.95	0.56-1.78	0.89	0.51-1.71
